# Andrews Bridge: The fixed but removable solution- A case report

**DOI:** 10.6026/973206300213852

**Published:** 2025-10-31

**Authors:** Chaithanya Ramisetty Nagabhushan, Srinidhi Bhat, Jaya Krishna T, Dimple M, Swathi K. Bhat

**Affiliations:** 1 Department of Prosthodontics, Sharavathi Dental College & Hospital, Shimoga, Karnataka, India

**Keywords:** Andrew's bridge, fixed-removable prosthesis, bars & sleeve attachment, Seibert's class III, esthetics

## Abstract

Rehabilitation of lost teeth especially in esthetic regions with compromised ridges is very unpredictable and challenging to the prosthodontist. Rehabilitating these patients with unaesthetic long span fixed partial dentures may be unacceptable to the patient. This case report presents rehabilitation of a 48 year old patient with missing mandibular anterior teeth and Seibert's class III using a Fixed-removable prosthesis (Andrews Bridge). Andrews's bridge system is mostly indicated in patients with large hard and soft tissue defects. This prosthesis helps in restoring the function, speech with improved esthetics.

## Background:

The fixed-removable type of prosthesis was introduced by Dr James Andrews. Andrews Bridge mainly consists of fixed and removable components. The fixed component mainly made up of crowns on the abutment teeth on either side of the defect joining through a cast or preformed metal bar and the removable component mainly comprises of a removable partial denture with a clip to seat on the metal bar [1]. The primary indication of this Fixed-removable prosthesis is defects with large hard and soft tissue loss which comes under Seibert's class III [2]. The Indications of fixed removable partial dentures are: Patients with opposing dentition restricting the esthetic placement of the pontics of a fixed partial denture, Patients needing diastema to harmonize the natural dentition, Patients who have extensive alveolar bone and soft tissue loss [3].

Seibert's classification for Residual ridge defects [4]

[1] Class I - Loss of buccolingual width with normal height of the ridge

[2] Class II - Loss of height with normal buccolingual width of the ridge

[3] Class III - Combination type defect (loss of both height and width of the ridge)

The incidence of Seibert's Class III defects are 56%, whereas class I defects are seen in 33% of the population and the least being occurrence of class II defects in 3% of the population [5, 6].

## Case report:

A 48 year old female patient reported to the Prosthodontics Department, with a chief complaint of missing teeth in the lower anterior teeth region and difficulty in speaking since 3 years with depression in the lower lip region. On intraoral examination, mandibular central and lateral incisors were missing with loss of residual ridge height and width. Various treatment options were explained to the patient, Andrews Bridge was considered as the most feasible option. Patient was explained about the procedure in patients own language and informed consent was taken.

## Procedure:

[1] Diagnostic impressions made and casts were prepared.

[2] Vital tooth preparation was done with respect to 33, 34, 43 and 44.

[3] Gingival retraction was done and a definitive impression was made using polyvinyl siloxane (Zhermack Hydrorise) impression material.

[4] Provisionalizations were done with Protemp - II (3M ESPE, USA) and were cemented on to the prepared teeth.

[5] Wax pattern was made on the prepared cast to incorporate the hader bar for the attachment of clip in the pontic or removable component of the prostheses.

[6] Metal try in was done to access the fit of the prosthesis, once after the metal trial was confirmed. Shade selection was done to match the metal ceramic fixed partial denture to the adjacent natural teeth. The final prosthesis was processed.

[7] The metal ceramic fixed partial denture with hader bar with adequate tissue relief was cemented and try in was done for the removable component.

[8] Patient's comfort, esthetics, phonetics, fit and retention were assessed and the final prosthesis was delivered to the patient.

[9] Post-operative instructions were given to the patient and patient was recalled for further follow up visits.

## Discussion:

The restoration of a large dentoalveolar defects are primarily carried out using removable partial dentures, especially in Seibert's class III where there is loss of both height and width of the tissues. However the removable partial dentures may be least retentive and unacceptable by the patient [7].

The prosthetic treatment options to restore dentoalveolar defects are,

[1] Conventional fixed partial dentures

[2] Implant supported prosthesis

[3] removable partial dentures

[4] Fixed-removable partial dentures [8].

The Andrews bridge system is a type of fixed-removable partial denture with a bar and sleeve which is firly attached to the abutment teeth to retain the flange of the removable component of the prosthesis [9]. Depending upon the area of the bar attachment Andrews bridge system is divided as pontic supported Andrews bridge and implant supported or bone anchored Andrews bridge [10]. Rathee *et al.* described the use of Neodynium Iron Boron magnets instead of bar and sleeve. This modification was mainly done to reduce the cost of the bar and the clip attachment. However some disadvantage of using intraoral magnets in oral environment can lead to corrosion of the magnets and loss in retention of the prosthesis, cytotoxic effects [11]. Advantages of the Andrews bridge system includes reduction in bulk of the denture, available of the prefabricated bar to follow the ridge contours, can replace up to four missing teeth, offers good retention of the denture to the underlying bar, offers high yield and tensile strength, it also allows to replace the lost tissue segment through the denture flange, Special transfer sleeves are available for each type of bar, making the fabrication of the removable component easier, improved esthetics, phonetics [12, 13]. Roy *et al.* [14] described the use of digital technology like computer-assisted designing/computer-aided manufacturing, and direct metal laser sintering has shown better predictable outcomes with reduced work flow. Chandra *et al.* emphasized the use of bone anchored or implant supported Andrews bridge, he also proposed that the use of Andrews bar is superior than that of implant supported fixed prosthesis and hence adding an option to improve patient's esthetics and comfort [15]. This fixed removable type of prosthesis was used to treat patients with generalized periodontitis, oroantral fistula and cleft lip and palate in adjunct to other surgical with successful outcomes as described by Sethna *et al.* [16] and Harish [17] *et al.* Many other authors have also described the use of different attachment systems to improve the retention of the removable prosthesis such as CekaPreci-Horix attachment with very predictable precision fit [18]. Hence, the fixed-removable prosthesis improved the well-being of the patients by improving the comfort of the patient.

## Conclusion:

Rehabilitation of large hard and soft tissue defects in the anterior region with Andrews's bridge system had shown good patient comfort, phonetics, esthetics. This prosthesis not only helps in replacing the missing teeth but also helps in replacement of lost tissue around it. The main disadvantage of this prosthesis is loss of retention with time, which can identified and corrected by replacing the clip component in the removable denture during the regular follow ups. Hence, this treatment modality is considered as an effective option in restoring the large intraoral defects.
